# Commercial resource potential of an invasive sea cucumber: nutritional analysis of *Synaptula reciprocans*

**DOI:** 10.7717/peerj.20466

**Published:** 2026-03-04

**Authors:** Mulkibar Ciftcioglu, Osman Sabri Kesbiç, Halit Filiz, Sevan Ağdamar

**Affiliations:** 1 Graduate School of Natural and Applied Sciences, Mugla Sıtkı Koçman University, Mugla, Turkey; 2 Department of Animal Nutrition and Nutritional Diseases, Kastamonu University Veterinary Faculty, Kastamonu, Turkey; 3 Faculty of Fisheries, Mugla Sıtkı Koçman University, Muğla, Turkey; 4 Department of Forestry, Bayramiç Vocational School, Canakkale Onsekiz Mart University, Turkey

**Keywords:** Sea cucumber, Nutritional composition, Bioinvasion, Mineral content

## Abstract

**Background:**

*Synaptula reciprocans*, originally native to the Indo-Pacific region, is widespread in the Red Sea. The species entered the Mediterranean during the 1970s and 1980s and has been reported along the Turkish coastline since 2003, where it has established invasive populations. European Union Regulation No. 1143/2014 encourages the investigation of alternative uses and the assessment of the economic potential of invasive species as part of management and control strategies. This study aims to evaluate the nutritional value and elemental composition of *S. reciprocans*, an invasive species in the Mediterranean, in order to assess its suitability for human consumption and potential alternative applications.

**Methods:**

*S. reciprocans* were sampled from two different stations (L1: Gokova Bay and L2: Gulluk Bay) over two seasons, as winter (S1) and summer (S2), and transported to the laboratory under cold chain conditions (+4 °C). Moisture, ash, crude protein, crude fat, fatty acid, amino acid, and elemental content analyses were performed using standardized methods, including gravimetric, Kjeldahl, GC/MS, LC/MS-MS, and ICP-OES techniques.

**Results:**

The findings indicate that both locality and season significantly influence the species’ nutritional properties (*p* < 0.05). Dry matter content exhibited considerable variation across seasons, with the highest concentration observed in L2/S2 and L2/S1. Crude protein levels peaked in winter across both regions (L2S1, L1S1), while fat content was consistently higher in Bodrum samples compared to Gokova (*p* < 0.05). Ash content was highest in Gokova across both seasonal periods (*p* < 0.05). The fatty acid profile demonstrated notable seasonal and regional differences (*p* < 0.05), with linoleic acid (C 18:2) emerging as the predominant polyunsaturated ω-6 fatty acid. Additionally, amino acid analysis revealed significant variation (*p* < 0.05), identifying alanine, asparagine, glutamine, and proline as the dominant amino acids. Elemental analysis highlighted the absence of copper (Cu) in all sampled tissues, while sodium (Na) was consistently the most abundant mineral.

## Introduction

Marine ecosystems face mounting challenges, including pollution, habitat destruction, loss of biodiversity, overfishing, and the spread of invasive species. One of the environmental challenges faced by these areas is the introduction of invasive species, which further aggravates existing issues. The frequency and magnitude of these introductions are steadily rising, posing escalating threats to ecosystems (Ricciardi et al., 2021). On the other hand, global climate change facilitates the spread of invasive species both directly and indirectly, allowing them to act as a driving force in biodiversity loss (Díaz & Malhi, 2022; Intergovernmental Science-Policy Platform on Biodiversity and Ecosystem Services (IPBES), 2023). Controlling of these species, which pose a critical challenge to biodiversity conservation and sustainable ecosystem management, often requires significant financial resources and complex management processes (Zenetos et al., 2010; Kleitou et al., 2021).

The European Union (EU) (2014) Regulation 1143/2014 on invasive species aims to control or eliminate priority species. In this context, EU Member States’ priority action plan for invasive species (Regulation 1143/2014) includes prevention, early detection, rapid eradication, and management measures. One of the strategies for mitigation is assessing the economic potential of invasive species. In Türkiye, which is not an EU member, the first legal regulation and action plan for invasive species was introduced for pufferfish (Türkiye Ministry of Agriculture and Forestry, 2024). This action plan includes measures such as incentives for catching invasive species (Kaplan, Yildirim & Yildirim, 2022) and/or commercial utilization strategies (Busch, Weimer & Keppner, 1999; Aydın, Düzgüneş & Karadurmuş, 2016; Demirel et al., 2021; Dağtekin, Ceyhan & Dağtekin, 2023). These practices suggest that economic utilization of invasive species can help limit their spread and mitigate their impact on ecosystem balance.

The Mediterranean Sea, with its unique geographical features, is home to a significant number of endemic species, comprising about 20% of its marine life (Garcia-Bustos, 2025). The opening of the Suez Canal in 1869 has particularly impacted the region, facilitating the introduction of non-native species from the Indo-Pacific, which has significantly altered both the ecological balance and economic structures (Galil, 2006; Çınar et al., 2011). The rapid increase in invasive species intensifies competition with native species, negatively affecting their populations (Antoniadou & Vafidis, 2009). Furthermore, these invasive species contribute to the damage of important marine habitats and ecosystems, disrupting food webs and ecosystem functions, and ultimately reducing the Mediterranean’s biodiversity (Galil, 2007; Sala et al., 2011; Katsanevakis et al., 2013; Bellard et al., 2016; Castro-Díez et al., 2019; Bédry et al., 2021; Tsirintanis et al., 2022). The disruption of ecosystem services also results in considerable socio-economic challenges, further exacerbating the problems caused by invasive species.

Sea cucumbers (Holothuroidea) are classified among the benthic fauna and serve as indicators of a healthy marine ecosystem due to their filtration capabilities (Martín et al., 2017; Parra-Luna et al., 2020). Namely, sea cucumbers are significant components of the benthic system due to their role in regulating sediment organic material and detrital dynamics (Purcell et al., 2016; Rakaj et al., 2023; Grosso et al., 2023). In addition to their ecological significance, they hold substantial commercial potential due to their high nutritional value, low fat content, and rich mineral composition (Chen, 2003; Wen, Hu & Fan, 2010). Due to their high nutritional value and rich biochemical composition, sea cucumbers are widely used in both gastronomy and health sectors, particularly in East Asian countries, where they are considered a luxury food item. These attributes have significantly increased their economic value (Fabinyi, 2012). These animals boast an impressive nutritional profile, featuring high-quality protein and lipids, along with essential vitamins such as Vitamin A, Vitamin B1 (thiamine), Vitamin B2 (riboflavin), and Vitamin B3 (niacin). Additionally, they are particularly rich in minerals like calcium, magnesium, iron, and zinc (Mamelona, Saint-Louis & Pelletier, 2010; Liu et al., 2021). These animals possess impressive components in terms of primary and secondary metabolites and offer a wide range of applications, whether as raw materials or as products and food items. For instance, primary and secondary metabolites, as well as bioactive compounds derived from these animals, exhibit a wide variety of therapeutic effects in humans, including antimicrobial, antioxidant, anti-cancer, anti-inflammatory, wound-healing, and immunomodulatory activities. Moreover, agents that inhibit the onset and progression of numerous pathogenic diseases can also be obtained from these organisms (Hashim, 2007; Senthilkumar & Kim, 2013; Chiaramonte et al., 2020; Luparello et al., 2019, 2022; Luparello, 2021; Liu et al., 2021). Owing to these remarkable attributes, sea cucumbers have garnered substantial attention in scientific research over recent years (Luparello et al., 2022).

The earliest records of *Synaptula reciprocans* (Forsskål, 1775) in the Mediterranean are known from the coasts of Cyprus and Israel (Cherbonnier, 1986; Galil, 2007). Subsequently, the species expanded its distribution and has been reported from the coasts of Lebanon, Syria, Greece, and Türkiye (Zibrowius & Bitar, 2003; Bitar, Dupuy de la Grandrive & Foulquie, 2003; Pancucci-Papadopoulou et al., 2005; Çınar et al., 2006; Galil, 2006; Koukouras et al., 2007; Antoniadou & Vafidis, 2009; Ragkousis et al., 2017). Regarding this invasive species, Ciftcioglu & Filiz (2021) and Ragkousis et al. (2017) reported interactions with protected local endemic species in the Mediterranean, including *Posidonia oceanica*, *Pinna nobilis*, and *Aplysina aerophoba*.

Therefore, carefully monitoring this invasive species and investigating alternative utilization methods are crucial in implementing measures to mitigate its impact on local species. However, in literature, research on this species has primarily focused on its taxonomy and distribution, while information regarding its nutritional composition, economic evaluation, and potential alternative uses remains highly limited. To mitigate the ecological pressure exerted by *S. reciprocans* and uncover its potential economic value, this study aims to analyze the species’ detailed nutritional content and explore alternative utilization possibilities.

## Materials and Methods

### *S. reciprocans* sampling

**Figure 1:**
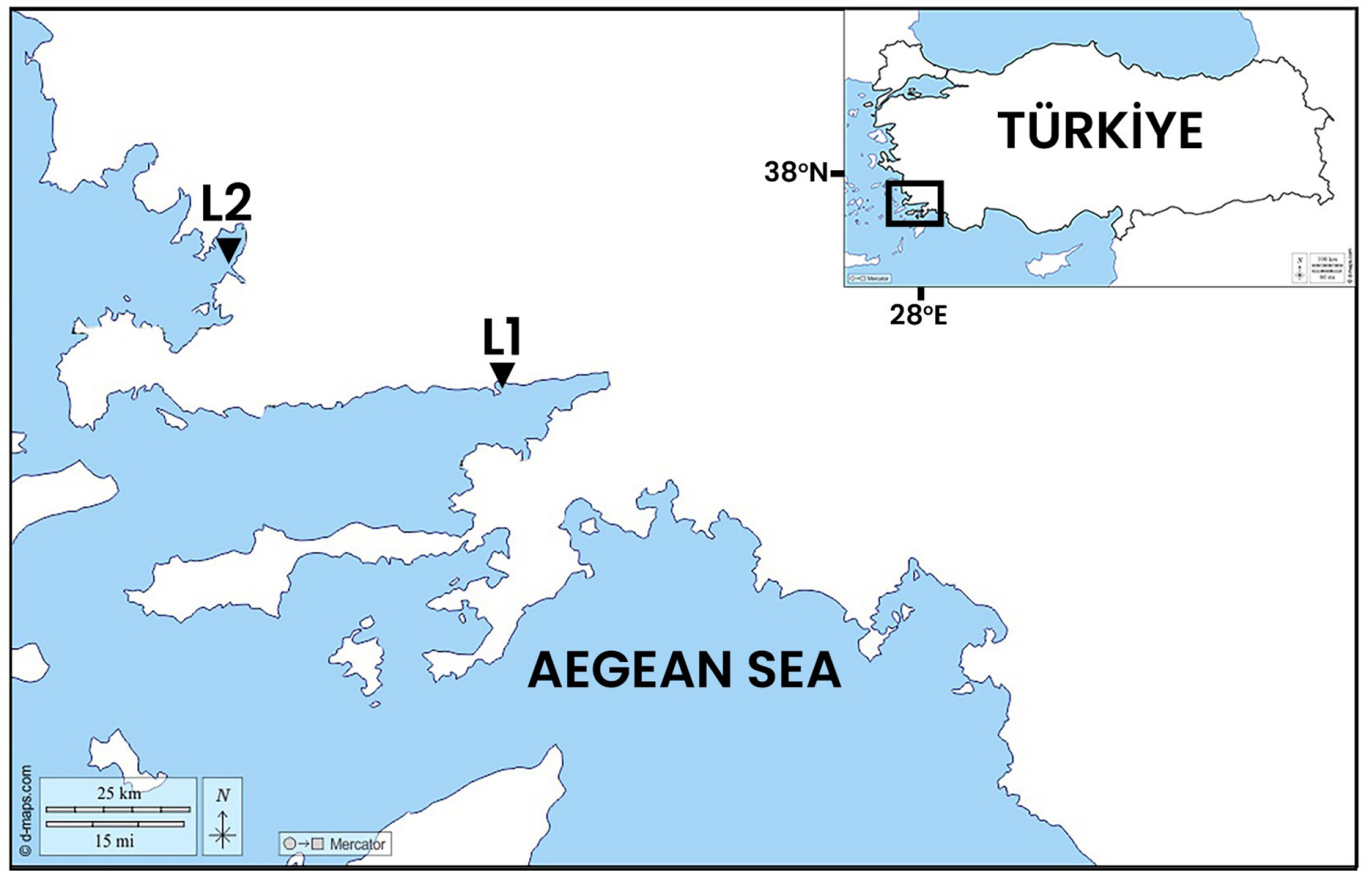
Sampling areas L1: Gokova Bay, L2: Gulluk Bay. Map source credit: https://www.d-maps.com/carte.php?num_car=274798&lang=en, Open Database License (ODbL).

The specimens of *S. reciprocans* were sampled (three individuals from each area per season, total 12 individuals). from Gokova and Gulluk Bays from the south Aegean Sea (Fig. 1) (coordinates; L1: 37°01′42″N 28°05′55″E, L2: 37°14′08″N 27°35′16″E). The samples were labelled for season (Winter (9^th^ December 2022), (S1), Summer (23^rd^ July 2022) (S2)) and location (Gokova Bay (L1), Gulluk Bay (L2)), then the samples were transported to the laboratory under a cold chain (+4 °C) condition.

### Moisture and ash analysis

Each specimen was initially weighed using sterilized and pre-tared petri dishes on a precision balance with an accuracy of 0.0001 g, without the removal of internal organs. Tissue isolation was subsequently performed by dissecting the specimens with plastic instruments. The isolated edible tissues were transferred into sterilized and pre-tared petri dishes, followed by final weighing and recording of the measurements. These tissues were then placed into individually labeled 20 ml tubes and subjected to a two-step freezing process: first at −20 °C for 24 h, and then at −80 °C for 48 h. Following freezing, the samples were lyophilized (freeze-dried) for 48 h. The dry weights of the samples were measured and recorded. Moisture and dry matter contents were calculated gravimetrically. All other nutritional analyses were carried out on those dried samples.

Ash content was determined *via* gravimetric analysis according to the standard method described by Kesbiç & Yigit (2019). For this analysis, dry tissue samples obtained from the moisture determination step were used. Porcelain crucibles were placed in a muffle furnace at 550 °C for 4 h until a constant weight was achieved. The crucibles were then transferred to a desiccator to cool. Empty crucibles were weighed using a precision balance with an accuracy of 0.0001 g, and their tare weights were recorded. Subsequently, 1 g of each sample was placed into the crucibles and incinerated at 550 °C until complete ash formation. After cooling in a desiccator, the crucibles containing ash were re-weighed. All measurements were performed in triplicate.

Specimens were weighed and processed under controlled conditions, including freezing, lyophilization, and dissection. Moisture and dry matter content were determined *via* gravimetric measurements, while ash content was quantified using standardized combustion techniques. The ash content was calculated using the formula: % Crude Ash = (Final Weight – Initial Weight)/Sample Weight × 100 (Kesbiç & Yigit, 2019).

**Crude protein analysis:** Crude protein analyses were performed based on the Kjeldahl method according to Kesbiç & Yigit (2019).

% Crude Protein = [14.01 × (A − B) × N]/W × 10 Where: A: Volume of titrated acid (ml), B: Volume of acid used for the blank test (ml), N: Normality of HCl, W: Sample weight (g).

The protein factor for animal products is 5.55, so the obtained %N value was multiplied by 5.55 to calculate the % crude protein. % Crude Protein = %N × 5.55.

**Crude lipid analysis:** Homogenized dry samples were subjected to solvent extraction using a methanol: chloroform mixture (1:2, v:v) and homogenized with an Ultraturrax. The extracted lipid fraction was separated *via* filtration, followed by evaporation using a rotary evaporator and subsequent drying in an oven at 60 °C. Precise weight measurements were recorded to determine crude lipid content. The lipid extracted from this procedure was further used for fatty acid composition assessment. All analyses were performed in triplicate to ensure accuracy and reproducibility. The crude lipid content of the sample was calculated using the formula:

% Lipid = [(Flask Tare + Lipid) – Flask Tare]/Sample Weight × 100 (Folch, Lees & Sloane Stanley, 1957).

**Fatty acid analysis:** A total of 20 mg sample extracted from crude lipid analysis was mixed with 1 mL of 1 N sodium hydroxide in methanol. The mixture was then heated at 80 °C for 15 min to facilitate saponification. The mixture was heated at 110 °C for 15 min to facilitate transesterification, after which 1 mL of 14% boron trifluoride in methanol was added. Subsequently, 1 mL of *n*-hexane was added, and the mixture was vortexed for 1 min to ensure thorough blending. Then, 3 mL of saturated sodium chloride solution was introduced, allowing the upper liquid phase to be collected as the sample solution. The fatty acid profile of the lipid samples was analyzed using a Shimadzu GC-MS QP 2010 ULTRA system (Shimadzu, Kyoto, Japan), ensuring precise identification and quantification of individual fatty acids. RTX2330 capillary column (60 m; 0.25 mm; 0.20 µm) was used in the system, with high-purity helium (99.99%) serving as the carrier gas to ensure analysis. The column furnace was maintained at 100 °C, while the injection and interface temperatures were both set at 250 °C. The ion source temperature was adjusted to 200 °C, with a system pressure of 90 kPa and an injection volume of 1 µL. The oven temperature program was as follows: an initial hold at 100 °C for 5 min, followed by a controlled increase at a rate of 4 °C per minute until reaching 240 °C, where it was sustained for 15 min (Kesbiç et al., 2023).

**Amino acid composition analysis:** To determine the amino acid profile, the samples were homogenized and then hydrolyzed under a nitrogen atmosphere at 110 °C for 24 h using 6 N HCl (0.1 g/20 mL) (Rodrigues et al., 2022). The analysis was carried out using the Shimadzu LC-MS 8040 device (Shimadzu Corporation, Kyoto, Japan) (Sun et al., 2016). For separation, the Zorbax Eclipse AAA column (4.6 × 150 mm, 3.5 μm) was used. During the chromatographic separation process, mobile phase A consisted of a mixture of formic acid and water at a 1:1,000 (v/v) ratio, while mobile phase B was a solution of formic acid and methanol at the same 1:1,000 (v/v) ratio (Kesbiç et al., 2024). The analysis was conducted at a flow rate of 1 mL/min, with an injection volume of 0.2 μL and a column temperature of 40 °C. The calibration for amino acid analysis was performed following the methods of (Roy et al., 2016), using a certified L-amino acid mixture standard (Sigma-Aldrich 79,248).

**Elemental content analysis:** The elements analyzed in this study were selected based on their nutritional, physiological, and ecological relevance to *S. reciprocans*, as well as their presence and significance in previous environmental studies conducted in the Gokova and Gulluk Bays (Çağlar, Aksu & Altuğ, 2020; İlhan, 2024). Macroelements (Na, K, Ca, Mg, P, S) were included due to their essential roles in osmoregulation, skeletal structure, and metabolic processes (Geng et al., 2016; Zaksas et al., 2022). Potentially toxic metals (Cd, Pb, As, Cr, Ni, Al) were selected as indicators of environmental pollution (Marrugo-Negrete et al., 2021), supported by regional studies showing moderate contamination levels in Gulluk Bay sediments. Trace and antioxidant elements (Fe, Mn, Zn, Se, Cu) were analyzed for their importance in enzymatic and cellular functions (Zaksas et al., 2022; Ye et al., 2025). Geochemical markers (Si, Ba, Au) were included to assess possible geological or anthropogenic signatures in the habitat (Marrugo-Negrete et al., 2021).

The sample quantity used was 0.25 mL, and the packaging sample contained 0.1 g of nitric acid. A total of 10 mL of 97% nitric acid was used for digestion. The microwave temperature program started at 200 °C, with an additional 10 mL of 97% nitric acid applied at 210 °C. The temperature was gradually increased to the target level. The standby time was 15 min, followed by additional hold times of 15 and 20 min. The maximum power applied during digestion was 1,400 W, maintained for 15 min (Kaya, Zurnacı & Şener, 2024).

The elemental composition of sea cucumbers was quantitatively analyzed in triplicate using the inductively coupled plasma-optical emission spectrometer (ICP-OES), which is widely used for the analysis of macro and micro trace elements (Douvris et al., 2023). Multi-element stock solution IV (Merck, Darmstadt, Germany) was used as the calibration standard. Standard calibration solutions were prepared by diluting the 1,000 ppm mixed standard stock solution IV. After selecting the calibration curve ranges (5–6 points), the prepared calibration solutions were analyzed using the SpectroBlue ICP-OES instrument. Linearity was checked, and detection limits were calculated as LOD (Limit of Detection) and LOQ (Limit of Quantitation).

The ICP-OES instrument used was the SPECTRO BLUE II model. Analyses were performed in triplicate. The spray chamber was cyclonic. The plasma segment operated in axial and radial modes. The TochBox temperature reached a maximum of 58 °C. The nebulizer flow rate was 0.8 L/min, and the nebulizer pressure ranged from 2.0 to 4.0 bar. The plasma torch was made of quartz. The gas used was argon with a purity of 99.9%. The main argon pressure was maintained between 6–8 bar. The coolant flow rate was 13 L/min, and the auxiliary gas flow rate was 0.8 L/min. The sample pump speed was 30 rpm, and the plasma power was set to 1,400 W.

**Statistical analysis:** The relationships between the analysis results obtained in the study were evaluated using one-way Analysis of Variance (ANOVA). Comparisons between groups were determined using the Tukey multiple comparison test. Statistical analyses were conducted using the SPSS 22.0 software and evaluated at a significance level of *P* < 0.05. Elemental concentration values were expressed as mean ± standard deviation (SD) based on triplicate measurements for each region and season. To evaluate data reliability and variability, the coefficient of variation (CV%) and the 95% confidence interval (CI) were calculated for each element within each group (L1S1, L1S2, L2S1, L2S2). The coefficient of variation was computed as the ratio of standard deviation to the mean, expressed as a percentage. The 95% CI was calculated assuming a normal distribution and *n* = 3.

## Results

The nutrient analysis conducted to determine the nutrient content of the species *S. reciprocans* is presented in Table 1. The findings clearly demonstrate that both the season and the region where the species was sampled had a significant effect on nutrient composition (*p* < 0.05). When examining the nutrient content, the dry matter content of *S. reciprocans* showed significant variation across all seasons (*p* < 0.05). The highest dry matter content was observed in individuals collected in L1S2 (12.20% ± 0.03). The second-highest dry matter content was found in samples collected in L2S1 (10.14% ± 0.01) (*p* < 0.05). The subsequent dry matter values were recorded in L1S1 and L2S2.

**Table 1:** Nutrient content of *S. reciprocans* (%).

RS/M	L1S1	L1S2	L2S1	L2S2
Dry matter	9.12 ± 0.02^c^	12.20 ± 0.03^a^	10.14 ± 0.01^b^	8.85 ± 0.10^d^
Crude protein	17.12 ± 0.09^b^	16.07 ± 0.20^c^	19.43 ± 0.38^a^	16.67 ± 0.34^b^
Crude fat	3.52 ± 0.27^c^	1.58 ± 0.20^d^	7.83 ± 0.07^a^	5.08 ± 0.18^b^
Crude ash	72.48 ± 0.19^a^	68.64 ± 0.31^b^	60.46 ± 0.61^d^	65.66 ± 0.88^c^

**Notes:**

RS, Regions and seasons; M, matter; L1, Gokova Bay; L2, Gulluk Bay; S1, winter; S2, summer.

All analyses were conducted with three replicates (*n* = 3), and the data are presented as mean ± standard deviation. Different superscript expressions indicate statistical significance (*p* < 0.05).

Crude protein content reached its peak during the winter period in both regions (in L2S1 17.12% ± 0.09, L1S1 19.43% ± 0.38). Significant differences were observed among the samples from L1S1, L1S2, and L2S1 (*p* < 0.05), while the protein values from L2S2 were similar to those from L1S2 (*p* > 0.05). In terms of lipid content, the samples collected from Bodrum displayed higher fat levels compared to those from Gokova (*p* < 0.05). Additionally, seasonal differences within the same region showed that winter samples consistently had higher fat content than summer samples (*p* < 0.05). Across all analyzed samples, Gokova (L1S1, L1S2, respectively 72.48% ± 0.19; 68.64% ± 0.31) exhibited the highest ash content values consistently in both seasonal periods. The ash content of the samples also varied significantly depending on both the season and the region of collection (*p* < 0.05).

The data regarding the fatty acid profile of the species is presented in Table 2. The analysis revealed that the season and the region where the species was sampled had significant effects on the changes in fatty acids (*p* < 0.05). Furthermore, among the polyunsaturated ω-6 group fatty acids, linoleic acid (C 18:2) was identified as the major fatty acid in each group. In the fatty acid profile of L1S2, fatty acids other than linoleic acid were below the detection limit. In L2S1, myristic acid (C 14:0), eicosatetraenoic acid (C 20:4 (ETA)), and eicosapentaenoic acid (C 20:5 (EPA)) were not detected. Regarding the fatty acid content of the L1S1, ETA was also found to be undetected.

**Table 2:** Fatty acid profile of *S. reciprocans* (%).

RS/FA Types	L1S1	L1S2	L2S1	L2S2
C 14:0	2.54 ± 0.31^b^	n.d	n.d	4.05 ± 0.50^a^
C 16:1	6.21 ± 1.65^a^	n.d	0.58 ± 0.17^b^	5.43 ± 1.32^a^
C 16:0	12.20 ± 2.67^a^	n.d	9.86 ± 1.98^a^	13.98 ± 1.87^a^
C 18:2	57.47 ± 8.08^b^	99.99 ± 0.01^a^	58.06 ± 9.06^b^	53.87 ± 4.45^b^
C 18:1	11.54 ± 2.29^b^	n.d	28.18 ± 6.60^a^	8.58 ± 1.26^bc^
C18:0	2.92 ± 0.68^b^	n.d	2.17 ± 0.49^ab^	3.46 ± 0.33^a^
C 20:4 n-3	n.d	n.d	n.d	2.23 ± 0.27
C20:4 n-6	2.65 ± 0.50^a^	n.d	1.14 ± 0.19^b^	2.46 ± 0.55^a^
C20:5 n-3	4.47 ± 1.18^a^	n.d	n.d	5.93 ± 1.65^a^

**Notes:**

RS, Regions and seasons, FA, fatty acid; L1, Gokova Bay; L2, Gulluk Bay; S1, winter, S2, summer, n.d, not detected.

All analyses were conducted with three replicates (*n* = 3), and the data are presented as mean ± standard deviation. Different superscript expressions indicate statistical significance (*p* < 0.05).

The amino acid profiles of the species sampled from different regions and seasons are presented in Table 3. The sampling time and region significantly influenced the amino acid profile of the species (*p* < 0.05). Furthermore, the major amino acids of the species were identified as alanine (Ala), asparagine (Asp), glutamine (Glu), and proline (Pro).

**Table 3:** Amino acid profile of *S. reciprocans* (%).

RS/Aa types	L1S1	L1S2	L2S1	L2S2
**Essential amino acids (EAAs)**				
Leu	4.51 ± 0.01^c^	4.84 ± 0.04^b^	5.16 ± 0.01^a^	3.85 ± 0.01^d^
Arg	2.06 ± 0.05^a^	1.84 ± 0.01^b^	2.03 ± 0.03^a^	1.54 ± 0.03^c^
his	0.78 ± 0.01^a^	0.79 ± 0.01^a^	1.01 ± 0.02^a^	0.61 ± 0.00^c^
ile	5.80 ± 0.04^c^	6.12 ± 0.06^b^	6.42 ± 0.08^a^	4.96 ± 0.01^d^
lys	0.82 ± 0.06^b^	0.96 ± 0.04^a^	1.05 ± 0.02^a^	0.80 ± 0.05^b^
met	1.24 ± 0.04^b^	1.27 ± 0.02^b^	1.50 ± 0.02^a^	1.00 ± 0.01^c^
thr	5.20 ± 0.05^c^	5.68 ± 0.08^b^	5.45 ± 0.30^bc^	6.38 ± 0.17^a^
val	4.04 ± 0.17^a^	4.15 ± 0.05^a^	4.36 ± 0.07^a^	3.58 ± 0.16^b^
**Others**				
Ala	14.93 ± 0.18^a^	13.94 ± 0.28^b^	13.74 ± 0.22^b^	14.12 ± 0.38^b^
Asp	12.46 ± 0.28^b^	12.73 ± 0.15^ab^	12.96 ± 0.09^ab^	13.75 ± 0.76^a^
cys	0.36 ± 0.03^b^	0.40 ± 0.01^ab^	0.41 ± 0.01^a^	0.37 ± 0.00^ab^
gln	0.94 ± 0.02^b^	0.95 ± 0.01^b^	1.14 ± 0.02^a^	0.82 ± 0.02^c^
glu	23.01 ± 0.15^b^	23.02 ± 0.14^b^	22.58 ± 0.46^b^	24.67 ± 0.19^a^
phe	2.20 ± 0.03^b^	2.41 ± 0.01^a^	2.42 ± 0.04^a^	1.98 ± 0.01^c^
pro	15.21 ± 0.42^a^	14.55 ± 0.16^a^	13.49 ± 0.07^b^	13.40 ± 0.21^b^
ser	5.32 ± 0.30^b^	5.19 ± 0.13^b^	4.99 ± 0.19^b^	7.24 ± 0.57^a^
tyr	1.06 ± 0.00^c^	1.09 ± 0.00^b^	1.24 ± 0.01^a^	0.89 ± 0.01^d^

**Notes:**

RS, Regions and seasons; Aa, amino acid; L1, Gokova Bay; L2, Gulluk Bay; S1, winter; S2, summer.

All analyses were conducted with three replicates (*n* = 3), and the data are presented as mean ± standard deviation. Different superscript expressions indicate statistical significance (*p* < 0.05).

The mineral contents obtained from the elemental analysis of *S. reciprocans*, collected from different regions and seasons, are presented in Table 4. The mineral content of *S. reciprocans* varied significantly across sampling regions (Gokova and Gulluk Bays) and seasons (winter and summer), with all major differences reaching statistical significance (*p* < 0.05). Sodium (Na) levels were highest in summer samples from Gokova Bay (L1S2: 8.92 g/kg), while the lowest concentration was observed in summer samples from Gulluk Bay (L2S2: 7.21 g/kg). Other macro minerals, including magnesium (Mg), potassium (K), and sulfur (S), also exhibited elevated concentrations in Gokova Bay during the summer. In contrast, calcium (Ca) reached its peak in winter samples from Gulluk Bay (L2S1: 2.63 g/kg), indicating a seasonal shift in mineral accumulation patterns. Microelement analysis revealed that iron (Fe), manganese (Mn), silicon (Si), and gold (Au) were significantly higher in winter samples from Gulluk Bay. Iron content was particularly notable, with the L2S1 sample reaching 52.11 mg/kg. Chromium (Cr) showed the highest concentration in summer samples from Gokova Bay (L1S2: 2,354.01 mg/kg), suggesting regional and seasonal influences on trace metal uptake.

**Table 4:** Mineral substance content of *S. reciprocans*.

RS/Min	L1S1	L1S2	L2S1	L2S2
g/kg				
Na	7.45 ± 0.04^b^	8.92 ± 0.05^a^	8.87 ± 0.01^a^	7.21 ± 0.08^c^
Mg	0.98 ± 0.00^b^	1.10 ± 0.02^a^	1.07 ± 0.00^a^	0.85 ± 0.01^c^
Ca	1.37 ± 0.02^d^	1.71 ± 0.05^c^	2.63 ± 0.05^a^	2.26 ± 0.04^b^
K	0.82 ± 0.01^c^	1.41 ± 0.01^a^	1.08 ± 0.01^b^	0.78 ± 0.01^d^
P	0.34 ± 0.00^c^	0.37 ± 0.00^a^	0.35 ± 0.00^b^	0.37 ± 0.00^a^
S	1.58 ± 0.01^b^	2.02 ± 0.03^a^	1.56 ± 0.01^b^	1.60 ± 0.00^b^
mg/kg				
Al	1.70 ± 0.04^c^	0.41 ± 0.07^d^	13.01 ± 0.27^a^	4.66 ± 0.17^b^
As	13.58 ± 0.20^ab^	13.42 ± 0.32^b^	13.74 ± 0.29^a^	14.18 ± 0.19^a^
Ba	0.03 ± 0.00^b^	n.d	0.15 ± 0.01^a^	0.04 ± 0.01^b^
Cd	0.34 ± 0.00^c^	0.39 ± 0.01^a^	0.35 ± 0.01^bc^	0.37 ± 0.01^ab^
Cr	2014.86 ± 9.15^c^	2354.01 ± 10.58^a^	2120 ± 30.10^b^	1803.00 ± 33.20^d^
Cu	n.d	n.d	n.d	n.d
Fe	21.04 ± 0.44^c^	14.03 ± 0.09^d^	52.11 ± 0.53^a^	26.13 ± 0.50^b^
Pb	6.56 ± 0.14^b^	7.06 ± 0.02^a^	6.93 ± 0.27^ab^	6.00 ± 0.06^c^
Mn	1.39 ± 0.03^c^	1.25 ± 0.02^c^	7.61 ± 0.12^a^	5.97 ± 0.09^b^
Ni	5.13 ± 0.05^a^	5.29 ± 0.08^a^	4.86 ± 0.05^b^	4.27 ± 0.12^c^
Se	10.06 ± 0.27	10.02 ± 0.07	10.41 ± 0.09	10.13 ± 0.18
Si	117.87 ± 1.01^b^	120.14 ± 0.94^b^	169.34 ± 2.72^a^	117.80 ± 2.94^b^
Zn	0.44 ± 0.01^c^	0.61 ± 0.01^a^	0.51 ± 0.00^b^	0.40 ± 0.01^d^
Au	6.05 ± 0.11^b^	6.50 ± 0.13^a^	6.64 ± 0.11^a^	5.78 ± 0.01^c^

**Notes:**

RS, Regions and seasons; Min, mineral; L1, Gokova Bay; L2, Gulluk Bay; S1, winter; S2, summer, n.d, not detected.

All analyses were conducted with three replicates (*n* = 3), and the data are presented as mean ± standard deviation. Different superscript expressions indicate statistical significance (*p* < 0.05).

Arsenic (As) and selenium (Se) levels remained relatively consistent across all samples, showing no significant variation by season or location. Copper (Cu) was not detected in any of the analyzed specimens.

These findings demonstrate that the mineral composition of *S. reciprocans* is influenced by both environmental and temporal factors. The elevated sodium levels observed in certain samples, particularly those from Gokova Bay in summer, highlight the need for careful monitoring of edible marine species.

## Discussion

The present study sheds light on the nutritional composition and potential utilization of *S. reciprocans*, an invasive marine species that has rapidly expanded its population along the Mediterranean coastline, particularly in areas such as Gokova and Bodrum. By investigating its nutrient profile across different seasons and regions, this research aims to mitigate ecological pressure while exploring the economic viability of this species.

This study evaluates the nutritional composition of the invasive sea cucumber species *S. reciprocans*, found along the coasts of Türkiye, with a focus on seasonal and regional influences. The results demonstrate significant variability in dry matter, crude protein, lipid, and ash contents across different sampling stations and seasons. The average dry matter content was 10.07 ± 1.31% (*p* < 0.05), with the highest values observed in L1S2 and L2S1. Compared to traded species such as *Holothuria tubulosa* and *H. poli*, which exhibit higher dry matter levels (16.19% and 22.03%, respectively) (Sicuro et al., 2012), *S. reciprocans* shows lower values, likely due to environmental, physiological, the genetic and reproduction cycle differences.

Protein content peaked in L2S1, with an overall average of 19.64 ± 1.52% (*p* < 0.05). These values are lower than those reported for *H. tubulosa*, *H. poli*, and *H. mammata* in previous studies (Sicuro et al., 2012; González-Wangüemert et al., 2018). Seasonal variation was evident, with winter samples from Bodrum showing the highest protein levels, suggesting increased metabolic activity during colder months. Additionally, studies on the nutritional composition of different sea cucumbers have emphasized that crude protein content can vary depending on region and season (*e.g*., Aydin et al., 2011; Sicuro et al., 2012; González-Wangüemert et al., 2018; Sales et al., 2023).

Lipid levels varied significantly, with L2S1 and L2S2 samples showing higher fat content than L1S2 and L1S1. Winter samples consistently exhibited elevated lipid levels, indicating potential energy storage mechanisms in response to seasonal stressors. The average ash content was 66.81 ± 4.39% (*p* < 0.05), ranging from 60.46 ± 0.61% in L2S2 to 72.48 ± 0.19% in L1S1. These values are comparable to those of *H. poli* and *H. tubulosa* (González-Wangüemert et al., 2018), though regional differences were observed, consistent with findings from other Mediterranean studies (Sicuro et al., 2012; González-Wangüemert et al., 2018). Overall, the study highlights the influence of environmental conditions, particularly seasonality and geographic location, on the nutritional profile of *S. reciprocans*. These findings contribute to a better understanding of the species’ ecological adaptability and potential for commercial utilization.

In this study, the fatty acid revealed that linoleic acid (C 18:2), a polyunsaturated ω-6 fatty acid, was identified as the dominant fatty acid in all groups. Linoleic acid is an essential omega-6 fatty acid and plays a role in various biological functions in the body. However, balancing the omega-6/omega-3 ratio is important for cardiovascular health (Simopoulos, 2002).

Öztürk & Gündüz (2018), in their study on the local sea cucumber (*H. tubulosa*), identified palmitic acid (C16:0) and eicosatrienoic acid (C20:3 n3) as the dominant fatty acids. In studies conducted on *H. tubulosa* (Aydin et al., 2011) and *Holothuria forskali* (Bilgin & İzci, 2016; Telahigue et al., 2014), arachidonic acid (C20:4 n6) was found to be the predominant fatty acid. According to these studies, the linoleic acid content in *H. tubulosa* and *H. forskali* is lower compared to *S. reciprocans* in terms of fatty acid profile. As linoleic acid is classified as an essential fatty acid in the human diet, this invasive species could serve as a valuable resource to produce dietary supplements. Further research is warranted to explore the potential applications of *S. reciprocans* in nutritional and health-related industries, leveraging its high linoleic acid content. The analysis of the fatty acid profile of *S. reciprocans* highlights the significant impacts of season and region on fatty acid composition (*p* < 0.05). These findings underscore the dynamic interplay between environmental conditions and the biochemical traits of the species. This approach could also contribute to managing the ecological impact of this invasive species.

Additionally, in samples collected from L1S2, other fatty acids were below the detection limit, highlighting a unique seasonal or regional influence that could warrant further investigation into lipid synthesis and utilization during warmer months (Zhang et al., 2017; Nishanthan et al., 2018). On the other hand, samples collected from L2S1 revealed the absence of certain fatty acids, including myristic acid (C 14:0), eicosatetraenoic acid (C 20:4, ETA), and eicosapentaenoic acid (C 20:5, EPA). This suggests seasonal environmental factors or physiological mechanisms that might suppress the synthesis or accumulation of these fatty acids in colder periods. In addition, certain fatty acids in sea cucumbers may not be detectable due to seasonal variations and/or analytical methods. For instance, Fawzya et al. (2015) did not detect C:14 fatty acid in their study on the fatty acid profile of sea cucumbers in Indonesia. Therefore, the absence of certain fatty acids in our study is considered natural and expected. Moreover, when reviewing existing research on *S. reciprocans*, it becomes evident that there is a lack of information regarding its nutritional composition and fatty acid profile. This study represents the first attempt to present such data for this species.

The results of the amino acid profile of the studied species revealed that the dominant amino acids are alanine (Ala), asparagine (Asp), glutamine (Glu), and proline (Pro). Furthermore, *S. reciprocans* is rich in branched-chain amino acids such as leucine, isoleucine, and valine. These amino acids play an important role in muscle protein synthesis and energy production (Hossain, Dave & Shahidi, 2020). Previous research on *H. tubulosa*, *H. poli*, and *H. mammata* species has reported that the most abundant amino acids are alanine, arginine, glutamic acid, and glycine (González-Wangüemert et al., 2018; Öztürk & Gündüz, 2018). This study identified alanine (Ala), asparagine (Asp), glutamine (Glu), and proline (Pro) as the major amino acids in the species. This consistent dominance of specific amino acids suggests their crucial roles in the physiological processes of *S. reciprocans*, such as protein synthesis, energy metabolism, and stress response mechanisms. The prominence of these amino acids aligns with their essential functions in supporting the survival and adaptation of organisms in varying environmental conditions. The amino acid profile of *S. reciprocans* reveals that proline is the most abundant amino acid. Proline is a major amino acid involved in collagen synthesis and stability, contributing directly to the structural integrity of skin, joints, and connective tissues. Given the presence of proline in *S. reciprocans*, its consumption may offer nutritional benefits relevant to skin and joint health (Meena, Mengi & Deshpande, 1999; Phang, Liu & Zabirnyk, 2010; Shaw et al., 2017; Li & Wu, 2018; Sionkowska et al., 2020). Mineral content and, particularly, heavy metal accumulation in species with filtration ability are important criteria for consumption in recommended species (Mohsen et al., 2019; Abugoufa et al., 2022). Therefore, in the present study, which investigates the nutritional composition and potential, the mineral composition of *S. reciprocans* was also analyzed, and the results highlight notable trends.

Sea cucumbers are generally rich in minerals such as Ca, Mg, Fe, and Zn (Bordbar, Anwar & Saari, 2011). González-Wangüemert et al. (2018) found high levels of Ca, Mg, and K in *H. poli*, whereas *H. tubulosa* contained elevated concentrations of Fe and Zn. However, in the current study, the mineral with the highest concentration in *S. reciprocans* was Na. The importance of sodium highlights its critical role in maintaining osmotic balance, nerve function, and other essential physiological processes in aquatic organisms (Yang et al., 2024). The homogeneity of sodium levels across sampling sites and seasons suggests that its availability and uptake are largely stable and not significantly affected by environmental changes, likely due to the species’ ability to effectively regulate sodium under varying conditions. Given the well-established link between high sodium intake and elevated blood pressure and cardiovascular risk (Yang et al., 2024), regular consumption of sea cucumbers with high sodium content may contribute to increased dietary sodium exposure, particularly in salt-sensitive populations. Therefore, monitoring and managing sodium levels in edible marine species such as sea cucumbers should be approached with caution, as they may pose a public health concern.

Additionally, a study conducted in the Mediterranean reported Se levels in the edible tissues of *H. poli* and *H. tubulosa* as 4.24 ± 0.04 mg/kg and 4.18 ± 0.16 mg/kg, respectively (Sicuro et al., 2012; Lin et al., 2018). In contrast, the present study found Se levels in *S. reciprocans* ranging from 10.02 ± 0.07 mg/kg to 10.41 ± 0.09 mg/kg. The upper daily intake limit for Se in the human diet is 70 µg (Elvevoll et al., 2022), and it plays a crucial role in supporting immunity, providing protection against viral infections, and influencing reproductive system function (Winther et al., 2020). As a result, previous studies on sea cucumbers (Venugopal & Gopakumar, 2017) and the current research indicate that these marine animals are excellent sources of Se compared to other seafood products.

The absence of detectable levels of copper (Cu) across all sampled tissues indicates that this mineral may not play a significant role in the physiological processes of the species or that it exists in concentrations below the detection limits of the analytical method employed. This observation warrants further investigation, particularly to explore potential environmental or biological factors contributing to this absence.

The results demonstrate the importance of elemental composition studies in understanding the nutritional and ecological dynamics of *S. reciprocans*. While sodium’s consistency hints at its physiological indispensability, the absence of copper suggests potential regional or ecological factors that may influence trace mineral uptake or accumulation. Future studies could focus on examining the bioavailability of other trace elements, as well as expanding the analysis to include potential environmental variables such as water salinity, temperature, or substrate composition, which could further explain the observed patterns in mineral content.

Determining the nutrient composition of a newly considered food source is essential to understanding its potential benefits and risks for human consumption or other organisms (Elvevoll et al., 2022). This analysis provides insight into the species’ nutritional value, identifying which essential nutrients it offers and whether it poses any toxic effects.

Wen & Hu (2010) assessed the nutritional quality and potential hazards of sea cucumbers, comparing the recommended consumption limits set by international authorities for a 100 g portion (Elvevoll et al., 2022). They evaluated 11 different sea cucumber species and identified these animals as good sources of Na, Cl, Mg, Ca, Fe, Cu, Se, and Cr minerals in terms of daily intake levels in the human diet (Wen & Hu, 2010). An important consideration in the recommendation of species with potential for consumption is heavy metal concentrations. Although maximum daily intake limits for Hg, Cd, As, and Pb have been established for many aquatic organisms, specific limits for Echinodermata have not been determined (Purcell et al., 2016; Lawson et al., 2021). Consequently, studies on this topic have compared the mineral content and limits of sea cucumbers to those set for other aquatic animals, particularly bivalve mollusks (Elvevoll et al., 2022). In the literature, this value for bivalves is 1.0 mg/kg (Lawson et al., 2021). In the present study, Cd concentration in *S. reciprocans* was found to be between 0.34 and 0.39 mg/kg. Therefore, as long as daily intake does not exceed recommended levels, consumption is not considered hazardous. However, the lack of data on this topic should not be overlooked.

As stated, the exposure limits for arsenic (As) in aquatic organisms within the human diet have not been established (Elvevoll et al., 2022). Studies on arsenic levels detected in sea cucumbers (dry weight basis) indicate that *H. poli* contains 33.3 ± 0.9 mg/kg (Rasyid, Yasman & Putra, 2021) and 22.9 mg/kg (Montero et al., 2021), *Cucumaria frondosa* has 3.6 ± 0.4 mg/kg (Song et al., 2020), and *H. tubulosa* registers 18.0 mg/kg (Montero et al., 2021).

In this study, *S. reciprocans* exhibited total arsenic concentrations ranging from 13.42 to 14.18 mg/kg. These levels are comparable to those reported in other holothurian species such as *Holothuria tubulosa*, which has been shown to accumulate up to 24.8 mg/kg (Montero et al., 2021). However, a major limitation of the present study is the lack of speciation analysis to differentiate between organic and inorganic arsenic forms. This distinction is critical from a toxicological perspective, as inorganic arsenic is significantly more toxic and associated with carcinogenic risks (European Food Safety Authority (EFSA), 2009). In marine organisms, particularly in echinoderms and mollusks, the predominant form of arsenic is often organic arsenicals (*e.g*., arsenobetaine and arsenosugars), which are generally considered non-toxic to humans (Francesconi, 2010). Nevertheless, without speciation data, it is not possible to reliably assess the human health risk or compare the measured values to regulatory safety thresholds for inorganic arsenic. Therefore, although the total arsenic levels observed do not immediately suggest a major toxicological concern, this study highlights the necessity of future research employing arsenic speciation techniques to better inform ecological and food safety evaluations.

The mineral contents detected in *S. reciprocans* samples exhibited significant variations depending on both geographic location (Gokova and Gulluk Bays) and seasonal changes. These findings suggest that environmental conditions and habitat characteristics play a crucial role in mineral accumulation.

Phosphorus (P) and sulfur (S) concentrations were also higher in summer samples, likely reflecting increased metabolic activity and protein synthesis in *S. reciprocans*. Sulfur is a fundamental component that provides both structural integrity and biochemical integrity to connective tissues such as cartilage and skin by participating in the structure of glycosaminoglycans such as chondroitin sulfate, forming disulfide bonds in proteins, and playing critical roles in antioxidant processes through methyl transfer (Komarnisky, Christopherson & Basu, 2003).

Among the micro minerals, aluminum (Al), iron (Fe), and manganese (Mn) levels were significantly higher in Gulluk Bay. This increase may be linked to anthropogenic pressures in the region, including metal accumulation from marinas and coastal tourism activities (Çağlar, Aksu & Altuğ, 2020). Similarly, elevated levels of potentially toxic elements such as chromium (Cr) and arsenic (As) may indicate industrial discharges and urban waste inputs. Echinoderms are known for their high capacity to bioaccumulate such metals (Walker et al., 2001).

The presence of heavy metals such as lead (Pb) and cadmium (Cd) is particularly concerning from an environmental risk perspective. Accumulation of these metals in organisms can lead to toxic effects on osmotic balance, enzymatic functions, and reproductive capacity (Rainbow, 2002). Notably, higher Cd levels detected in summer samples from Gokova Bay may point to localized pollution sources.

Trace elements such as zinc (Zn) and selenium (Se) are vital due to their roles in antioxidant defense systems (Ferenčík & Ebringer, 2003). The lack of significant seasonal variation in Se levels suggests that this element is regulated homeostatically.

The detection of gold (Au) is noteworthy, as this element is typically present in marine environments only in trace amounts. The observed values may be associated with the region’s geological structure or anthropogenic contamination (Jones, 1970).

In conclusion, the mineral profile of *S. reciprocans* reflects the sensitivity of echinoderms to environmental variability and their potential for biological accumulation. These findings support the use of this species as a biomonitor and highlight its relevance as a bioindicator in regional pollution assessments.

The nutritional profile of a food source includes components such as proteins, fats, carbohydrates, vitamins, and minerals. These values help assess whether the species supports healthy growth and development in consumers. Furthermore, analyzing its effects on health can reveal functional benefits, such as anti-aging properties or immune system support, through the presence of antioxidants, specific vitamins, and minerals. Elemental analysis also plays a crucial role in determining whether the species contains heavy metals. If detected, it is vital to assess whether the concentrations fall within essential mineral limits or reach toxic levels.

Assessing the nutritional content of a species benefits both consumers seeking a healthy diet and the food industry in product development and marketing. With this vision, the conducted research lays the foundation for understanding the nutrient profile of an invasive sea cucumber species found in Turkish waters, facilitating future investigations and product development efforts.

## Conclusions

This is the first comprehensive study reporting the nutrient and mineral composition of the invasive sea cucumber species *S. reciprocans*, which is rapidly spreading along the Mediterranean coast. The data obtained indicate that the species can be utilised in the food and nutraceutical sectors as it is rich in linoleic acid, contains high levels of proline and other essential amino acids, and exhibits a remarkable profile of minerals such as sodium, magnesium and selenium. This potential could contribute to the control of invasive populations through targeted commercial fishing and reduce competitive pressure on native species. However, such a strategy should be planned within the framework of ecosystem-based management principles, as there may be negative impacts such as degradation of bottom habitats, bycatch risks and sudden changes in population dynamics. Therefore, the commercial exploitation of *S. reciprocans* should be limited in terms of fishing season, depth and method, supported by long-term monitoring programmes, and the biochemical data obtained should be integrated into decision-making mechanisms to ensure both biodiversity conservation and economic sustainability. However, the limitations of this study include the fact that some components in fatty acid analysis were below the detection limit, some amino acids such as glycine, which are critical in the structural proteins of the species, were not measured and the number of samples was relatively low. In future studies, it is recommended that a full amino acid profile (including glycine and hydroxyproline) and arsenic speciation analyses be performed with larger sample sizes, and that more sensitive analytical methods for fatty acid determination be used to identify low abundance components. Such improvements would improve the accuracy and reliability of the data obtained and allow a more comprehensive assessment of the ecological, nutritional and commercial potential of *S. reciprocans*.

## Supplemental Information

10.7717/peerj.20466/supp-1 Supplemental Information 1 Raw Data.

## Additional Information and Declarations

### Data Availability

The following information was supplied regarding data availability:

The raw measurements are available in the Supplemental File.
